# Future perspectives: United Kingdom National Multidisciplinary Guidelines

**DOI:** 10.1017/S0022215116000682

**Published:** 2016-05

**Authors:** E V King, K Harrington

**Affiliations:** 1 Consultant Head and Neck Surgeon Poole Hospital NHS Foundation Trust and University of Southampton, Southampton, UK; 2 Targeted Therapy Team, The Institute of Cancer Research, London, UK

## Abstract

The multidisciplinary management of head and cancer has changed radically in the last decade. This paper provides a glimpse of the emerging surgical and oncological interventions that may play major roles in the treatment paradigms of tomorrow.

## Surgery

Advances in surgical techniques appear slow and cumbersome compared with the rapid unravelling of molecular mechanisms responsible for head and neck squamous cell carcinoma (SCC). It is now clear that head and neck SCC can be sub-divided into two main cohorts, those that are driven by human papilloma virus (HPV), and those that are not. This is only the first level of patient stratification on our way to personalised medicine. However, it provides a sensible basis for trial recruitment, which is a good starting point.

It is clear that the majority of HPV-driven head and neck SCC patients do well, irrespective of treatment. This is in stark contrast to those patients who have developed a tumour secondary to tobacco and alcohol use, who may only have a 5 per cent survival advantage compared to those patients treated over 20 years ago with the same disease. Clearly, this heterogeneous group deserves better outcomes, not just in terms of survival, but also functional outcome improvement.

Trials now capture data relating to swallow (i.e. PATHOS in HPV-positive disease), revealing the change in concern regarding treatment-associated morbidity. Ideally, this would also be reflected in ongoing national data collection, formerly Data for Head and Neck Oncology (DAHNO), to be replaced by Head and Neck Cancer Audit (HANA).

Despite the incidence of some head and neck SCCs decreasing over time (i.e. larynx), these numbers are outweighed by the continued increase in other sub-types, specifically oropharynx (Cancer Research UK). This undoubtedly has economic implications as combined head and neck clinics see more patients year on year, often without additional clinical support. It is predicted that by 2020 there will be more cases of HPV-driven head and neck SCC than cervical cancer.^1^ Will the prophylactic HPV vaccine slow the trend? Despite not being introduced to address head and neck SCC, it undoubtedly should have an effect. There are ongoing discussions regarding vaccinating boys, to fall in line with Australia, the USA, Austria and parts of Canada, in view of the strong male predisposition to HPV-driven head and neck SCC. The arguments not to vaccinate boys relate to the short-term cost implications, which in many countries has been shown to be unsubstantiated,^2^^–^^4^ and the absence of evidence of efficacy of the vaccine against oropharyngeal disease.

Surgical instruments have continued to evolve and many teams use lasers and harmonic scalpels routinely. Some units use robotic surgery as part of their surgical approach, with an evolving body of non-randomised data to support its use.^5^^,^^6^ Long-term functional outcome data are still lacking with this new technology, but should be collected as a matter of course to ensure both survival outcomes and functional morbidity continue to improve.

Novel tools to improve the certainty of surgical resection margins intra-operatively are available in theatre, ranging from the use of Lugol's Iodine (LIHNCS – Lugol's Iodine in Head and Neck Cancer Surgery) to commercially available systems (PENTAX i-SCAN™, OLYMPUS narrow band imaging and STORZ spies™). Cutting edge molecular diagnostic tools (iKnife) require prospective data collection to support their use, but if confirmed may revolutionise the need for intra-operative frozen sections, with significant cost-saving associations.^7^

Advances in microvascular techniques push the limit of surgical resections, maximising the chances of surgical clearance and the associated links with improved survival. In addition, microsurgical techniques are being employed to reduce tumour- and/or treatment-associated morbidity, examples including complex nasal, midface and mandibular reconstruction, most of which benefit from computer-aided planning to fit individual defects and laryngeal re-innervation.

Imaging modalities continue to evolve and help facilitate patient selection and operative limits. Recent results from the positron emission tomography-Neck trial will undoubtedly influence neck management and future surveillance imaging, but this will require backing from the Royal College of Radiologists to support the change in clinical practice. Other avenues that may provide enhanced imaging techniques include dual-energy computed tomography (CT).^8^

Finally, surgeons are well placed to talk to patients about research trials, even if it is just a matter of taking consent to send tumour to the tissue bank. Research is fundamental to improve our understanding and treatment of this disease.

## Oncology

There have been significant recent advances in non-surgical oncological management of head and neck SCC. These developments are likely to continue to shape our thinking over the next decade as we develop more effective, less toxic treatments for head and neck SCC. The key themes are discussed below.

### Improved radiotherapy (RT) techniques

In comparison with treatment techniques that would have been standards of care one or two decades ago, the current routine daily practice of RT is completely unrecognisable. Three-dimensional conformal RT and intensity-modulated radiotherapy are now considered to be gold-standard treatments and are available in almost every centre in the UK. Even these approaches are being refined further with increasing application of image-guided RT. This involves using imaging investigations performed during a course of treatment to allow oncologists to adapt the RT plan to ensure adequate coverage of target volumes that contain (or may contain) cancer cells while, at the same time, sparing normal tissues. Increasing availability of linear accelerators with on-board cone-beam CT and technologies that allow fusion of planning CT scans with diagnostic magnetic resonance imaging scans will continue to drive this process. In addition, the development of newer technologies, such as the MR-Linac and proton beam therapy, means that the next decade is likely to see significant advances in the therapeutic index of RT.^9^^,^^10^

### Development of molecularly targeted radiosensitisers

As a result of meta-analyses of a large number of small- to medium-sized randomised trials, we now recognise RT delivered with concomitant platinum monotherapy as a standard of care for unresected, locally advanced head and neck SCC. A molecularly targeted antibody against epidermal growth factor receptor (EGFR), cetuximab, has also been shown to enhance the effect of RT in a single-phase III trial, but it did not yield additional benefit when combined with platin-based chemoradiotherapy.^11^ In the next decade, we are likely to see a number of new targeted radiosensitisers developed for use in patients with head and neck SCC. Improved understanding of key molecular events in the response of cancer cells to radiation has highlighted potential targets for developing tumour-selective radiosensitisers. In particular, dysregulated cell cycle control and/or loss of key components of the DNA damage response represent a molecular ‘Achilles’ heel' that is vulnerable to therapeutic exploitation with new agents that include poly ADP ribose polymerase, Chk1, poly ADP ribose polymerase and Wee1 kinase inhibitors.^12^

### Development of immuno-oncology (I-O) agents

In recent years, I-O has emerged as a major new modality in the treatment of many solid cancers, including head and neck SCC. This advance has been underpinned by huge strides in our understanding of the fundamental biological principles that guide the activity of the immune system. In particular, specific immune checkpoints have been discovered that are central components of normal immune responses. In health, such checkpoints function to inhibit T cells and prevent their chronic activation or misdirection against normal tissues. Effectively, they function as negative regulators or ‘brakes’ on the normal immune response. Many cancers subvert these inhibitory pathways in order to escape from immunosurveillance by activating brakes on the immune system. Immune checkpoint inhibitors are able to release these brakes on the immune system and trigger dramatic antitumour responses. Antiprogrammed death (PD)-1 and anti-PD ligand-1-targeted monoclonal antibodies have already shown activity in head and neck SCC^13^ and it is highly likely that other, newer checkpoint-inhibiting drugs will enter clinical practice in the next 5–10 years. It is very probable that head and neck SCC will continue to represent a promising target for such drugs.

### Development of effective adjuvant therapies

Previous attempts to use adjuvant chemotherapy in patients who had completed definitive treatment for locally advanced head and neck cancer were focused on cytotoxic chemotherapy and failed to demonstrate any benefit. Subsequent research moved towards assessment of small molecule inhibitors of growth factor receptors. Recent data have shown that the dual EGFR/human epidermal growth factor2 inhibitor, lapatinib, does not improve outcomes of post-operative chemoradiotherapy in patients judged to be at high risk of disease recurrence.^14^ In ongoing studies, the irreversible inhibitor of EGFR, HER2 and HER4, afatinib, is being tested as an adjuvant therapy in high-risk patients after definitive chemoradiation (phase III LUX2 study NCT01345669) or after post-operative chemoradiotherapy (GORTEC 2010-02, EudraCT 2010-023265-22). The increased prominence of I-O agents (vide supra) signifies that adjuvant trials of such agents will be conducted in the coming years. Hopefully, such studies will deliver a successful outcome against locoregional and metastatic recurrence of head and neck SCC.

### Personalised treatment through molecular classification

We have made major advances in our understanding of cancer by examining the genetic nature of the disease [The Cancer Genome Atlas Network, 2015^15^]. Recent reports have provided detailed analysis of the mutational landscapes in different types of tumours and this work is beginning to provide insights that are likely to guide future therapeutic innovation. For example, the basis of the biological differences between HPV-positive and -negative cancers is clear when examining their different genetic profiles. The preponderance of inactivating events (mutations, epigenetic silencing) in the p53 pathway in HPV-negative disease contrasts strongly with the frequency of wild-type (i.e. normal) p53 in HPV-positive disease. In addition, specific abnormalities (e.g. PIK3CA mutations) are more common in HPV-positive disease and may be suitable targets for the specific drug therapies. It is likely that we will see further subcategorisation of head and neck SCC in the next decade and that will be accompanied by personalisation of treatment for individual patients based on the genetic content of their disease.

### Key points

• Increased patient participation in clinical trials
• Patient stratification for personalisation of treatment
• Improved imaging techniques
• Advances in surgical tools including robotic surgery
• Improved outcomes through new radiotherapy technologies
• Incorporation of immuno-oncology agents in radical and palliative treatment approaches
• Development of effective post-radiotherapy/chemoradiotherapy adjuvant treatments
